# A Path Model for Burnout in Community Mental Health Professionals

**DOI:** 10.3390/ijerph18189763

**Published:** 2021-09-16

**Authors:** Jin-Joo Chang, Sung-Hee Shin

**Affiliations:** College of Nursing Science, Kyung Hee University, Seoul 02447, Korea; jinjoo.j.chang@gmail.com

**Keywords:** burnout, emotions, fatigue, satisfaction, mental health

## Abstract

The purpose of this research is to identify a path model to explain burnout in community mental health professionals based on the compassion satisfaction–compassion fatigue (CS-CF) model. A total of 125 mental health professionals, including nurses, social professionals, and psychologists working in mental health welfare centers in various regions across South Korea were surveyed using a structured questionnaire. A path analysis was conducted using SPSS 24.0 and AMOS 24.0. The results showed that compassion satisfaction and compassion fatigue are significant predictors of burnout (β = −0.20, *p* = 0.011; β = 0.40, *p* < 0.001, respectively). The indirect pathways associated with burnout included occupational stress (β = 0.21, *p* = 0.021) and experience with aggressive behavior in the workplace (β = 0.33, *p* = 0.004) through maladaptive cognitive emotion regulation and compassion satisfaction. The total effect of the variables on burnout via compassion fatigue and compassion satisfaction explained 62.5% of burnout among mental health professionals. These findings indicate that providing nursing interventions might reduce compassion fatigue and increase compassion satisfaction to reduce burnout. Furthermore, intervention programs that help to reduce the use of maladaptive cognitive emotion regulation strategies are necessary to effectively reduce burnout in mental health professionals.

## 1. Introduction

The enactment of the revised Mental Health Welfare Act has highlighted the importance of mental health promotion services for psychiatric patients in South Korea. In addition, this act has contributed to increased awareness of mental illness and recognition of the importance to prevent, treat, and manage mental illness in addition to hospitals [1]. In particular, the role of community mental health professionals, who are mental health welfare center practitioners providing mental health promotion services within the community, has been emphasized [1,2]. Community mental health professionals include mental health nurses, social professionals and psychologists educated for more than 1 year at a training institution designated by the Ministry of Health and Welfare [2].

According to a 2018 survey that examined the working conditions of mental health professionals at the Seoul Mental Health Welfare Center, the average length of employment was 3 years, which is shorter than the 6.5-year average length of employment for all South Korean workers in 2018 [3,4]. The short duration of employment and the elevated number of turnover factors for community mental health professionals, responsible for the public’s mental health were found to be associated with burnout [5]. Burnout is defined as physical, emotional, and mental exhaustion that occurs following repeated and long-term exposure to stress in the workplace [6]. Such burnout is commonly reported by professionals working in mental health facilities [5].

Burnout is primarily caused by occupational stress related to the work environment [5,6], which occurs when the environment is not suitable [6]. Occupational stress is caused by work-related changes in an individual’s physical, physiological, and psychological conditions due to the environment and conditions they experience in the course of performing their roles. Burnout caused by occupational stress is commonly found in people whose job is to help others, such as community mental health professionals. People whose job involves helping others are placed in an environment where they must provide professional and moral care simultaneously with human empathy. Therefore, people with such jobs continue to experience occupational stress and eventually develop burnout [7]. Community mental health professionals may experience burnout due to overlapping workplace roles, peer conflict, excessive workload, and employment insecurity [5]. They often provide services under poor working conditions and insufficient staffing [2,5]. This environment increases the likelihood of mental health professionals becoming unable to provide high-quality services [5].

Burnout may also occur in one’s interpersonal environment, including psychological pressure from interpersonal relationships and secondary trauma stress from vicarious experiences [8]. A survey of employees at the Korea Mental Health Welfare Center reported that 63.6% of respondents experienced verbal threats from clients, 34.5% experienced threats from client guardians, 33.2% experienced physical assault from clients, and 27.3% experienced emotional sequelae following a client’s suicide [2]. Among the job responsibilities of community mental health professionals are the identification and registration of clients, home visits for individual case management, and emergency services, which expose them to violent behavior, delusions, and other acts of aggression in interpersonal relationships [2]. After experiencing symptoms following aggressive behavior from a client in the workplace, mental health professionals may avoid situations reminiscent of the attack and show symptoms similar to post-traumatic stress disorder (PTSD), including nightmares and insomnia. In severe cases, negative sequelae include burnout, depression, or suicide [9].

Not all professionals display symptoms (e.g., PTSD) after experiencing such an attack [10]. The symptoms of the attack experience are determined by variables corresponding to their personal environment following the experience [10]. As service providers, such as community mental health professionals, sympathize with clients’ trauma, they experience cognitive changes and the fatigue process—the process of becoming victims themselves [10]. Thus, it is critical for mental health professionals to recognize and control negative emotions they experience after facing aggressive behavior in the workplace, which requires cognitive emotional regulation strategies and the cognitive control of information that emerges during the experience of a stressful event [11]. Cognitive emotional regulation strategies can also be a way for individuals to choose how to control their emotions and solve problems in certain situations that stimulate emotional response [11].

With the increasing importance of balancing the positive and negative outcomes of individuals’ choices in the working process, the concepts of compassion fatigue and compassion satisfaction emerged [8,12]. Both compassion fatigue and satisfaction contribute to understanding and confirming employee burnout by proving an integrated perspective, and research has confirmed their relevance to burnout [8]. The compassion satisfaction–compassion fatigue (CS-CF) model is a subjective assessment of the possible factors experienced by professional care providers whose job responsibilities involve working to assist others [12]. In this model, the variables theorized to precede burnout, compassion satisfaction, and compassion fatigue are divided into work, client, and personal environments [12]. Compassion fatigue refers to the negative phenomenon experienced by professional care providers while taking care of people who have experienced stressful events [12]. Increased compassion fatigue has a negative impact on physical and mental health [8,12]. This can lead to increased burnout, which can again result in poor quality patient care [8,12]. By contrast, professional care providers sometimes feel positive emotions when helping others, which are called compassion satisfaction, and this leads to positive rewards that reduce burnout [12].

Research has been conducted on compassion fatigue, compassion satisfaction, and burnout among nurses and doctors who may experience aggressive behavior during work [8]; however, thus far, no research on this topic among community mental health professionals exists, to the best of our knowledge. Previous studies on community mental health professionals included those on the relationship between ordinary job-related variables and the factors influencing them [5], qualitative research on burnout [5], or those limited to trauma-related variables without specific mediating variables [10].

The current study attempted to develop a path model that could explain the pathways to burnout among community mental health professionals, based on the CS-CF model, which might provide insight into reducing burnout by identifying predictor variables, including occupational stress (work environment), experiences with aggressive behavior in the workplace (client environment), and cognitive emotional regulation strategies as input (personal environment). Ultimately, this study attempts to develop a theoretical foundation for human resource management that promotes harmony between service providers and clients and ensures high-quality service by supporting the psychological well-being of community mental health professionals.

Based on existing research, this study tests the CS-CF model to identify the pathways leading to burnout in a sample of community mental health professionals, which might verify the model’s suitability and identify factors contributing to burnout directly and indirectly.

### 1.1. Study Purpose

Based on Stamm’s CS-CF model [12], this study aimed to explain mental health professionals’ burnout as being a result of compassion satisfaction and compassion fatigue, considering the associations of the work, client, and personal environment.

### 1.2. Conceptual Framework

**Figure 1:**
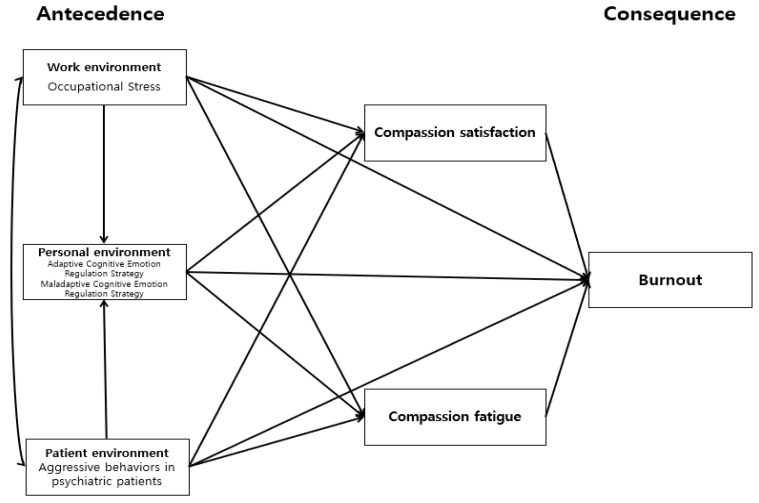
Conceptual framework.

The CS-CF model, including predictor and outcome variables, and the resulting variables that contribute to compassion satisfaction and compassion fatigue, represents a subjective framework for understanding burnout among professional care providers [12]. In this model, the variables proposed to determine burnout, compassion satisfaction, and compassion fatigue were divided into work, client, and personal environments [12]. In the CS-CF model’s conceptual framework, the contributing factors are associated with the process, which, in turn, associates the results, and the impact connects in one direction. Stamm et al. [12] proposed that the three environments would have a negative effect on compassion fatigue and burnout, and a positive effect on compassion satisfaction. The authors further identified the working environment as an environment for work-related care providers. This study defines the working environment as an occupational stressor that occurs when one’s working environment is unsuitable for an individual’s motivation or ability, or when an individual’s ability is ill-suited for the working environment [6]. Client environment is an environment for caring for clients arising from interactions between care providers and clients [12]. In this study, the client environment was conceptualized as the clients’ verbal, nonverbal, and physical behaviors threatening community mental health professionals, other people, or property during the provision of service [12,13]. The personal environment is that of the care providers [12], and in this study, it was operationalized as the cognitive emotional regulation strategies following stressful events experienced by community mental health professionals [11]. The personal environment could be associated with related variables even when individuals are exposed to the same working and client environments [10]; thus, the working and client environments are associated with the personal environment in this study. The study was designed to complement the CS-CF model to understand the variables leading to burnout (Figure 1).

## 2. Materials and Method

### 2.1. Research Design

This cross-sectional survey aimed to identify pathways from occupational stress, aggressive behavioral experiences, cognitive emotion regulation strategies, compassion fatigue, and compassion satisfaction to burnout.

### 2.2. Research Participants and Data Collection

The study participants were community mental health professionals from 11 mental health welfare centers across South Korea who had worked for at least 3 months [3], understood the study’s purpose, and agreed to participate in the study.

The data were collected from 31 July to 9 September 2017. Permission to participate in the survey was obtained via phone and online from community mental health professionals from 11 mental health welfare centers across South Korea. Once permission was obtained, each mental health welfare center was visited to explain the research objectives to community mental health professionals with over 3 months of work experience and who agreed to complete the questionnaire.

The number of participants in the path analysis should be at least 5 to 10 times the number of estimated parameters. In this study, 18 parameters were estimated; thus, the number of required participants was calculated to range between 90 and 180 [14]. Consequently, 150 questionnaires were distributed, 140 were returned, and 125 were included in the final analysis. Fifteen questionnaires were excluded because of unfaithful answers, and they were considered incomplete.

### 2.3. Measures

#### 2.3.1. Occupational Stress

The instrument assessing occupational stress included 24 questions rated on a 4-point scale, ranging from 1 = not at all to 4 = very much. Total scores ranged from 24 to 96, with a higher score indicating a higher degree of occupational stress. Example questions for the occupational stress subscale included the following: “I am instructed to work in an inconsistent or non-standard state”; “The future is uncertain due to unstable situation in the center”; and “I can adjust my workload and schedule by myself”. The internal consistency of the original scale had a Cronbach’s α of 0.82 [15], and in this sample, it was 0.96.

#### 2.3.2. Aggressive Behaviors in Psychiatric Patients

The scale used to measure participants’ experiences with aggressive behavior in the workplace included 16 questions. Respondents were asked to indicate their experience with each aggressive event described in a question using either 0 = no or 1 = yes, with scores ranging from 0 to 16. Higher scores indicated a greater degree of experience with different aggressive behaviors. Example questions regarding experience with aggressive behavior in the workplace subscale included the following: “Some clients exhibit actions such as hitting the head or fist against the wall or throwing things, etc.”; “Some clients inflict severe damage to a person (e.g., fracture)”; and “Some clients are angry and swear severely or use language threatening to others”. The internal consistency of the original tool had a Cronbach’s α of 0.87 [16], and in this study, Cronbach’s α was 0.87.

#### 2.3.3. Adaptive Cognitive Emotion Regulation Strategy

The adaptive cognitive emotion regulation strategy was measured using 20 questions, rated on a five-point Likert scale ranging from 1 (not at all) to 5 (very much), with total scores ranging from 20 to 100. Higher scores indicated a higher degree of adaptive cognitive emotion regulation strategies. Example questions for the adaptive cognitive emotion regulation-strategy subscale included the following: “I think of the best way to deal with the situation”; “I look for the positive aspects of the problem”; and “I think there is something to learn from that situation”. In a study by Kim [17], in terms of Cronbach’s ⍺, the internal consistency of the tool ranged from 0.66 to 0.85; in this study, the internal consistency was expressed by a Cronbach’s ⍺ of 0.89.

#### 2.3.4. Maladaptive Cognitive Emotion Regulation Strategy

The maladaptive cognitive emotion regulation strategy was measured with 16 questions rated on a five-point Likert scale ranging from 1 (not at all) to 5 (very much), with total scores ranging from 16 to 80. Higher scores indicated a higher degree of maladaptive cognitive emotion regulation strategies. Example questions for the maladaptive cognitive emotion regulation-strategy subscale included the following: “I think it is my fault”; “I think others are wrong about that”; and “I keep thinking about how terrible the situation was”. In a study by Kim [17], the internal consistency of the tool was indicated by a Cronbach’s ⍺ ranging from 0.53 to 0.78, and in this study, the internal consistency was expressed by a Cronbach’s ⍺ of 0.91.

#### 2.3.5. Compassion Satisfaction

Compassion satisfaction was measured using items from the Professional Quality of Life Scale (ProQOLS) developed by Stamm [12], after receiving approval from the developers of the Korean version. The 10 items related to compassion satisfaction were rated on a five-point scale from 1 (not at all) to 5 (very much), with total scores ranging from 10 to 50. Higher scores indicate higher compassion satisfaction. Example questions for the compassion satisfaction subscale included the following: “I feel satisfied with the fact that I can help others”; “I feel satisfied with the fact that I can help others”; and “I am proud that I can help others”. The internal consistency of the original tool was expressed by a Cronbach’s ⍺ of 0.88 [12], and it was 0.90 in this study.

#### 2.3.6. Compassion Fatigue

Compassion fatigue was measured using items from the ProQOLS after receiving approval from the developers of the Korean version. Compassion fatigue was assessed using 10 relevant items from the scale, and items were rated on a five-point scale from 1 (not at all) to 5 (very much), with total scores ranging from 10 to 50. Higher scores indicate higher compassion fatigue. Example questions for the compassion fatigue subscale included the following: “I feel like I am experiencing the trauma someone I have helped to has undergone” and “I avoid certain situations or activities that remind me of the frightening experiences the people I have helped to have undergone”. The internal consistency of the original tool was expressed by a Cronbach’s ⍺ of 0.81, and it was 0.89 in this study.

#### 2.3.7. Burnout

The burnout tool was measured using items from the ProQOLS after receiving approval from the developers of the Korean version. Ten questions related to burnout from the overall scale were rated on a five-point scale from 1 (not at all) to 5 (very much), with total scores ranging from 10 to 50. Higher scores indicate higher levels of burnout. Example questions for the burnout subscale included the following: “I am happy or I am unhappy”; “I am “inhibited” by the system”; and “I feel trapped in my job as a caregiver”. The internal consistency of the original tool was expressed by a Cronbach’s ⍺ of 0.75 [12], and it was 0.84 in this study. All measures reported in this study were used after receiving approval from their developers and translators [11,12,15,16,17,18].

### 2.4. Data Collection and Ethical Consideration

Data collection was approved by the bioethics review committee of Kyung Hee University in Seoul (IRB NO: Kyung Hee University KHSIRB-17-049) and was conducted with consent and cooperation from the team leaders at mental health and welfare centers nationwide, after they were contacted and informed about the purpose of the study. Before completing the questionnaire, participants were provided with a written explanation describing the purpose of the study and the post-participation benefits, the protection of participants’ confidentiality, anonymity, and the right to withdraw from the study. The explanation also stated the data would not be used for any purpose other than research. All participants provided written informed consent, considering the ethical aspects of the questionnaire. In addition, compensation was provided after the completion of participation in the study (a product some $10 in worth).

### 2.5. Data Analysis

Data were analyzed using SPSS 24.0 (IBM, Madison Avenue, New York, USA) and AMOS 24.0 (IBM, Madison Avenue, New York, USA). First, the differences between participants’ demographic characteristics and burnout were compared using frequency and percentages, *t*-tests, and analyses of variance. Post-hoc group differences were confirmed using Scheffé’s method. The mean and standard deviations for occupational stress, experience with aggressive behavior in the workplace, adaptive and maladaptive cognitive emotion regulation, compassion satisfaction, compassion fatigue, and burnout were calculated. Next, bivariate Pearson’s correlations were calculated between occupational stress, experience with aggressive behavior in the workplace experience, adaptive and maladaptive cognitive emotion regulation, compassion satisfaction, compassion fatigue, and burnout. Finally, a path analysis was conducted to examine the effects of occupational stress, experience with aggressive behavior in the workplace, adaptive and maladaptive cognitive emotion regulation, compassion satisfaction, and compassion fatigue on burnout in a sample of community mental health professionals.

## 3. Results

In total, 150 questionnaires were distributed, of which 140 were collected, with a response rate of 93.3%. After excluding 15 questionnaires with insufficient responses, 125 were used for the final analysis.

### 3.1. Participant’s Demographic Characteristics and Burnout

Burnout significantly differed between participants according to their age group *F ratio* *(F)* = 3.00, *p* = 0.034; ≤25 years: *Mean (M) =* 2.69, *Standard Deviation* (*SD) =* 0.70; 26–30 years: *M =* 2.91, *SD =* 0.58; 31–35 years: *M =* 2.62, *SD =* 0.61; >35 years: *M =* 2.54, *SD =* 0.57. However, the Scheffé test post-hoc results showed no significant group differences.

### 3.2. Occupational Stress, Experience with Aggressive Behavior in the Workplace, Cognitive Emotion Regulation Strategies, Compassion Satisfaction, Compassion Fatigue, and Burnout

Participants’ mean occupational stress was 50.50 (*SD =* 17.12) out of 100, with the mean score for “experiences with aggressive behavior in the workplace” being 7.59 (*SD =* 3.92) out of 16. Of the cognitive emotion regulation strategies, mean adaptive cognitive emotion regulation was 68.46 (*SD =* 9.35) out of 100, and the mean maladaptive cognitive emotion regulation was 47.63 (*SD =* 10.33) out of 80. The mean compassion satisfaction was 35.46 (*SD =* 5.93) out of 50, and mean compassion fatigue was 28.04 (*SD =* 7.17) out of 50. The mean burnout was 27.20 (*SD =* 6.10) out of 50 (Table 1).

### 3.3. The Relationship between Occupational Stress, Experience with Aggressive Behavior in the Workplace, Cognitive Emotion Regulation Strategy, Compassion Satisfaction, Compassion Fatigue, and Burnout in Community Mental Health Professionals

Correlations between burnout, occupational stress, experience with aggressive behavior in the workplace, cognitive emotion regulation strategy, compassion satisfaction, and compassion fatigue among participants are shown in Table 2. Burnout in community mental health professionals was positively correlated with occupational stress (*r* = 0.63, *p* < 0.001), maladaptive cognitive emotion regulation (*r* = 0.71, *p* < 0.001), experience with aggressive behavior in the workplace (*r* = 0.62, *p* < 0.001), and compassion fatigue (*r* = 0.73, *p* < 0.001). Since the correlation coefficient between occupational stress and experience with aggressive behavior in the workplace (*r* = 0.86, *p* < 0.001) was >0.8, multicollinearity between the variables was confirmed. As a result of the verification, the tolerance limit was not <0.1, and the variance inflation factors (VIF) value was >10. Furthermore, the association analysis using Cook’s Distance found that no object out of 125 was ≥1.0. This was followed by a residual analysis of the linearity of the model, the normality of the error term, and homoscedasticity.

### 3.4. Path Analysis of Burnout

**Figure 2:**
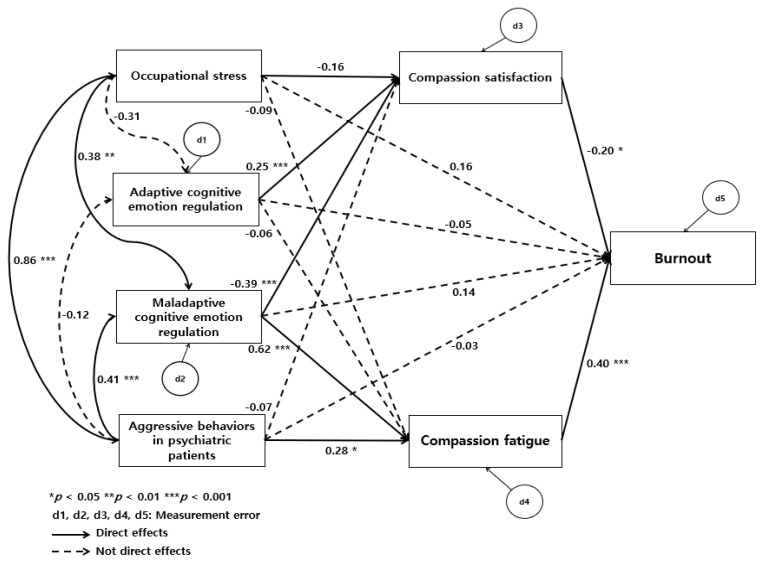
Testing for hypothetical model.

A path analysis was first conducted for the hypothetical model proposed in this study to check the mediating effects of adaptive and maladaptive cognitive emotion regulation strategies on paths from occupational stress, experience with aggressive behavior in the workplace, compassion satisfaction, and compassion fatigue leading to burnout. The fit of the model was assessed by χ^2^, *df*, Comparative Fit Index (CFI), Goodness of Fit Index (GFI), Normed Fit Index (NFI), Tucker Lewis Index (TLI), and root mean square error of approximation (RMSEA) using path analysis and maximum likelihood. The goodness-of-fit for the hypothetical model in this study was shown as χ^2^ = 0.99 (*p* = 0.320), *df* = 1, CFI = 1.00, GFI = 0.99, NFI = 0.99, TLI = 1.00, and RMSEA = 0.00, indicating an acceptable fit. The normality of the measurement variables was confirmed by examining the skewness and kurtosis of each measurement variable. Skewness values were determined to be outside normality at the *p* < 0.05 significance level if the value was greater than the absolute value of 3; further, kurtosis values were outside of normality when the absolute value reached 8 or 10 [14]. Therefore, the skewness and kurtosis of the variables in this study were within the ±2 absolute value range, indicating that they did not deviate from the assumption of normal distribution. The significance level was calculated from path estimates to clarify the path parameters and effectiveness in this study. The path loadings are shown in Table 3 and Figure 2, centered around the standardized path parameters.

The direct, indirect, and total effects of the measured pathways in the model suggest the mediating effects of adaptive and maladaptive cognitive emotion regulation on the paths from occupational stress, aggressive behavior, compassion fatigue, and compassion satisfaction on burnout in community mental health professionals. These paths and effects were as follows: occupational stress was not directly associated with burnout (β = 0.16, *p* = 0.157); however, it was indirectly associated with it (β = 0.21, *p* = 0.021). Although experience with aggressive behavior in the workplace was not directly associated with burnout (β = −0.03, *p* = 0.819), it was indirectly associated with it (β = 0.33, *p* = 0.004). Adaptive cognitive emotion regulation strategies were not directly associated with burnout (β = −0.50, *p* = 0.423) nor did it have an indirect effect (β = −0.07, *p* = 0.05). Although maladaptive cognitive emotion regulation strategies were not directly associated with burnout (β = −0.03, *p* = 0.197), they had an indirect effect (β = 0.32, *p* = 0.004). Although occupational stress did not have a direct effect on compassion satisfaction (β = −0.15, *p* = 0.237), it had an indirect effect via maladaptive cognitive emotion regulation strategies (β = −0.22, *p* = 0.008). Although occupational stress did not have a direct effect on compassion fatigue (β = −0.09, *p* = 0.432), it had an indirect effect via maladaptive cognitive emotion regulation strategies (β = 0.25, *p* = 0.004). Although experience with aggressive behavior in the workplace did not have a direct effect on compassion satisfaction (β = −0.06, *p* = 0.626), it had an indirect effect via maladaptive cognitive emotion regulation strategies (β = −0.18, *p* = 0.004). Experience with aggressive behavior in the workplace had a direct effect on compassion fatigue (β = 0.27, *p* = 0.014) and an indirect effect via maladaptive cognitive emotion regulation strategies (β = 0.26, *p* = 0.004). Lastly, compassion fatigue, maladaptive cognitive emotion regulation, compassion satisfaction, and adaptive cognitive emotion regulation strategies were associated with the total effect on burnout. The total effect of the variables on burnout via compassion fatigue and compassion satisfaction explained the 62.5% of burnout among mental health professionals.

## 4. Discussion

This study attempted to identify a path model to explain burnout in community mental health professionals based on the CS-CF model and recognized variables that might reduce it. Burnout was found to significantly differ across age groups and was high in those aged between 26 and 30 years; however, Scheffé’s post-hoc test showed no significant differences between the groups. Jo and Kim [8] reported that age was inversely correlated with the level of burnout, suggesting that older adults were less likely to experience professional burnout due to unfamiliarity with their work because they had more experience than younger workers. In this study, as for the ratios of community mental health professionals, regarding their age, 8.0% were younger than 25, 40.0% were aged between 26 and 30 years, and 52.0% were 31 or older; thus, the high degree of burnout in those aged 26 to 30 was consistent with Jo and Kim’s findings [8].

Similarly, Lee and Kim [19] analyzed burnout among psychiatric nurses using the instruments included in this study and reported that burnout was higher in those younger than 30 compared to those over 50, which was consistent with our results. Since community mental health professionals aged between 26 and 30 years were primarily responsible for practical tasks, such as weekly program management and visiting services for case management, working environment factors were likely to associate their burnout [3].

The mean burnout among the participants in this study was 27.2. Stamm et al. [12] examined burnout in a sample representing a variety of professions that included nurses in the United States and reported a mean score of 25, which is lower than that reported in this study. Furthermore, the mean burnout score in this study was also higher than that reported by Lee and Kim [19], who measured burnout using the Korean version of the ProQOLS developed by Stamm et al. [12], in a sample of full-time nurses who worked for over a year in psychiatric wards at university hospitals, psychiatric specialty hospitals, and psychiatric clinics. This is likely to be higher in all three, that is, nurses, social professionals, and clinical counselors, who were mental health professionals, rather than those who only worked in hospitals, given the unique nature of the work. Therefore, future research that compares burnout across various regions, occupations, and types of job responsibilities is necessary to gain a broader understanding of burnout and to separate and analyze variables related to its severity, such as environmental and personal characteristics.

In this study, burnout was significantly and positively correlated with occupational stress, experience with aggressive behavior in the workplace, maladaptive cognitive emotion regulation strategies, and compassion fatigue, and negatively correlated with adaptive cognitive emotion regulation and compassion satisfaction. Jang and Kim [20] further examined compassion satisfaction, compassion fatigue, and coping methods in a sample of emergency room nurses and reported that compassion satisfaction and compassion fatigue were associated with participants’ coping methods, consistent with this study. The current findings are also consistent with those of Kim and Yeom [21], who showed that compassion fatigue and compassion satisfaction in nurses were the primary burnout predictors. The current results are consistent with those from studies that included samples of nurses, elementary school teachers, play therapists, and emergency medical professionals [12,20,21,22,23]. Taken together, occupational stress, experience with aggressive behavior, maladaptive cognitive emotion regulation strategies, compassion fatigue, adaptive cognitive emotion regulation strategies, and compassion satisfaction are burnout-related factors.

Considering the variables that could be associated with burnout in community mental health professionals, compassion fatigue was observed to have the greatest positive effect, and compassion satisfaction the largest negative effect. In other words, the greater the compassion fatigue and the lower the compassion satisfaction, the higher the burnout experienced by participants. In addition, Park and Chun [8] reported that compassion satisfaction in child and youth welfare professionals had a negative effect on burnout, whereas compassion fatigue had a positive effect. They [8] also described subcategories of compassion fatigue, such as PTSD and vicarious trauma. Specifically, aspects of PTSD that encompass emotional aspects such as negative emotions, constraints, threats, and irritations received from clients, and experiencing clients’ vicarious trauma, appeared to contribute to negative emotions in the relationship with clients [10]. Thus, it is critical to check variables that can promote compassion satisfaction, a positive factor, and decrease compassion fatigue, a negative factor.

This study demonstrated that occupational stress, which is considered an aspect of the working environment, had no direct effect on burnout, compassion satisfaction, or compassion fatigue; however, it had an indirect effect on both compassion satisfaction and compassion fatigue through maladaptive cognitive emotion regulation strategies. In turn, maladaptive cognitive emotion regulation strategies, compassion satisfaction, and compassion fatigue were shown to have indirect effects on burnout. Previous research has demonstrated that community mental health professionals experience occupational stress, and ultimately burnout, due to workplace conflicts arising from overlapping roles within multidisciplinary teams across different occupations, identity confusion, lack of systematic education, and job insecurity [5]. In a qualitative study of mental health social professionals, Jung [23] reported that participants working at mental health sites were found to have residual occupational stress in their daily lives, which helped them avoid interpersonal relationships. As Jung’s research [23] was qualitative, direct comparison with this study, which is a quantitative one, might be problematic. However, this study only validated the indirect effects of occupational stress on burnout, rather than the direct effects. The results showed that occupational stress was associated with compassion satisfaction, compassion fatigue, and ultimately burnout through maladaptive cognitive emotion regulation, suggesting that maladaptive cognitive emotion regulation was a mediating variable.

In this study, experience with aggressive behavior, the variable assessing the client environment, was only directly associated with compassion fatigue, and was not directly related to compassion satisfaction or burnout. However, it had indirect effects on compassion satisfaction and compassion fatigue through maladaptive cognitive emotion regulation strategies. Furthermore, maladaptive cognitive emotion regulation strategies, compassion satisfaction, and compassion fatigue had indirect effects on burnout. Jung’s study of mental health social professionals [23] reported that participants faced threatening situations, including verbal aggression, physical aggression, sexual violence, and intimidation, while providing services at mental health sites. In addition, secondary traumatic stress experienced due to clients, compassion satisfaction, and compassion fatigue were identified in a wide range of interpersonal service professionals, such as doctors, mental health professionals, health care professionals, childcare professionals, and social professionals [23], consistent with this study. Numerous studies have examined the personal and internal responses of professionals after traumatic experiences [22]. The mediating association of maladaptive cognitive emotion regulation strategies, even following traumatic experiences, such as mental health professionals’ experiences with aggressive behavior in the workplace, suggests the possibility of having a healthy and positive well-being without showing adverse consequences, such as PTSD.

Adaptive cognitive emotion regulation strategies, which were measured constructs related to the personal environment of community mental health professionals, did not have direct or indirect effects on burnout, and maladaptive cognitive emotion regulation strategies were not directly associated with burnout but indirectly via compassion satisfaction and compassion fatigue. Maladaptive cognitive emotion regulation strategies had indirect effects on burnout through occupational stress, representing the participants’ working environment, and their experience with aggressive behavior in the workplace, an aspect of the client environment. Increased occupational stress and more numerous experiences with aggressive behavior in the workplace contributed to the use of more maladaptive cognitive emotion regulation strategies. Such strategies contributed to lowered compassion satisfaction and increased compassion fatigue, ultimately leading to higher burnout. These pathways are consistent with previous research showing that internal, individual protective factors mediate burnout, rather than external factors such as the working environment [22,23]. Im [22] suggested that cognitive emotion regulation strategies in workers were a critical factor that could influence burnout through self-compassion. The results presented by Im [22] are consistent with those in our study, indicating that cognitive emotion regulation strategies that balance emotions and cognition are critical to the overall delivery of client services, as well as burnout among community mental health professionals.

Maladaptive emotion regulation strategies that had indirect effects on burnout consisted of self-criticism about one’s experiences, blaming others for one’s experiences, rumination (i.e., thinking about feelings and accidents associated with static events), and destructive thinking, emphasizing the terrifying aspects of the experience. A study on the relationships between nurses’ experiences of traumatic events, cognitive emotion regulation strategies, and post-traumatic growth demonstrated that adaptive and maladaptive cognitive emotion regulation strategies had significant effects on post-traumatic growth [24]. The current findings also indicate that maladaptive cognitive emotion regulation strategies are a notable mediating variable in the theoretical burnout model for community mental health professionals, which is consistent with the mediating associations of trauma following experiences with aggressive behavior experienced during client service. A study of emergency room nurses by Jang and Kim [20] showed that their active responses, such as active behavioral coping and positive reinterpretation, negatively affected their compassion fatigue, whereas passive responses such as emotional expression and passive withdrawal affected it positively. Although this study did not assess mental health professionals’ responses, the present findings could be interpreted similarly as cognitive emotion regulation strategies were ways of coping with situations using either adaptive or maladaptive cognitive emotion regulation. Numerous studies have examined the mediating association of hospital conditions with nurses [20,21]; however, little research has examined how the working environment of community mental health professionals mediates burnout. Thus, additional research, similar to this study, is needed with samples of community mental health professionals to understand the indirect effects on burnout via compassion fatigue and compassion satisfaction, taking into account the aspects of the working, client, and personal environments in multidimensional ways.

This study suggests that cognitive emotion regulation strategies, seen as an aspect of community mental health professionals’ personal environment, represent a variable mediating burnout. Therefore, it is necessary to identify medium-and long-term policies that might ameliorate and manage burnout in community mental health professionals by actively utilizing psychological and emotional development programs, such as cognitive therapy, to reduce maladaptive cognitive emotion regulation. Specifically, programs should be developed that can help individuals who rely on inefficient regulation strategies, such as self-criticism, blaming others, constant thinking about feelings and accidents related to negative events, and emphasizing the fear of experience, to cope with conflict.

This study had several limitations. First, it did not consider various factors that could affect burnout among community mental health professionals. Second, it analyzed a path model for burnout that did not separate participants by specific occupation (e.g., mental health nurses, mental health social welfare professionals, and mental health clinical counselors). Third, in-depth analysis by occupation, and considering the characteristics of each job, will be required in the future. Finally, the study method requires discretion when attempting to generalize the results of this study. In addition, this study used a convenience sampling method to select mental health professionals working at mental health promotion centers throughout South Korea as participants; thus, further studies that include more widespread research sites and diverse participant groups are needed. This study also built on the cross-sectional data. The evidence to support this model based on the current cross-sectional data may be insufficient to draw causality, and more studies, including longitudinal and intervention studies, are needed to confirm the model.

## 5. Conclusions

This study attempted to test a path model based on the CS-CF model to understand burnout among community mental health professionals and provide a basis for identifying potential mediating variables that could reduce it. The results showed that the most influential variables for burnout in this sample of community mental health professionals were compassion fatigue and compassion satisfaction. Maladaptive cognitive emotion regulation strategies were not found to have a direct effect on burnout; however, they had an indirect effect through both occupational stress, representing the working environment in this study, and experience with aggressive behavior, representing the client environment. These results suggest that the correct use of cognitive emotion regulation strategies within the personal environment might act as a mediating variable that could reduce burnout. Therefore, given the current results, developing regular management programs whose effectiveness can be verified might reduce and prevent burnout in community mental health specialists, particularly those who address the mediating association of maladaptive cognitive emotion regulation.

## Figures and Tables

**Table 1:** Level of occupational stress, experience with aggressive behavior in the workplace, adaptive cognitive emotion regulation, maladaptive cognitive emotion regulation, compassion satisfaction, compassion fatigue and burnout in community mental health professionals (*N* = 125).

Variables	*M* (*SD*)	Min	Max	Range	Skewness	Kurtosis
Occupational stress	50.50 (17.12)	18	79	0–100	−0.04	−1.50
Experience with aggressive behavior in the workplace	7.59 (3.92)	0	16	0–16	0.07	−1.05
Adaptive cognitive emotion regulation strategy	68.45 (9.35)	43	91	20–100	−0.41	0.22
Maladaptive cognitive emotion regulation strategy	47.63 (10.33)	27	68	16–80	0.10	−1.01
Compassion satisfaction	35.46 (5.93)	23	50	10–50	0.22	−0.19
Compassion fatigue	28.04 (7.17)	12	44	10–50	−0.09	−0.53
Burnout	27.20 (6.10)	15	43	10–50	0.19	−0.62

*M* = Mean, *SD* = Standard Deviation, Min = Minimum, Max = Maximum.

**Table 2:** Correlations among occupational stress, adaptive cognitive emotion regulation, maladaptive cognitive emotion regulation, experience with aggressive behavior in the workplace, compassion satisfaction, compassion fatigue and burnout in community mental health professionals (*N* = 125).

Variables	1	2	3	4	5	6
	*r* (*p*)	*r* (*p*)	*r* (*p*)	*r* (*p*)	*r* (*p*)	*r* (*p*)
**2**	−0.42 (<0.001)	1				
**3**	0.73 (<0.001)	−0.51 (<0.001)	1			
**4**	0.86 (<0.001)	−0.39 (<0.001)	0.73 (<0.001)	1		
**5**	−0.59 (<0.001)	0.53 (<0.001)	−0.66 (<0.001)	−0.57 (<0.001)	1	
**6**	0.62 (<0.001)	−0.44 (<0.001)	0.79 (<0.001)	0.67 (<0.001)	−0.58 (<0.001)	1
**7**	0.63 (<0.001)	−0.46 (<0.001)	−0.71 (<0.001)	0.62 (<0.001)	−0.63 (<0.001)	0.73 (<0.001)

1 = occupational stress, 2 = adaptive cognitive emotion regulation strategy, 3 = maladaptive cognitive emotion regulation strategy, 4 = experience with aggressive behavior in the workplace, 5 = compassion satisfaction, 6 = compassion fatigue, and 7 = burnout.

**Table 3:** Effect coefficient for hypothetical model.

Endogenous Variable	Exogenous Variables	Direct Effect β (*p*)	Indirect Effect β (*p*)	Total Effect β (*p*)	SMC *
Burnout	Compassion satisfaction	−0.02 (0.011)	-	−0.20 (0.031)	0.63
	Compassion fatigue	0.40 (<0.001)	-	0.40 (0.005)	
	Adaptive cognitive emotion regulation	−0.05 (0.423)	−0.07 (0.050)	−0.13 (0.168)	
	Maladaptive cognitive emotion regulation	0.14 (0.197)	0.33 (0.004)	0.47 (0.004)	
	Occupational stress	0.16 (0.157)	0.21 (0.021)	0.37 (0.008)	
	Experience with aggressive behavior in the workplace	−0.03 (0.819)	0.33 (0.004)	0.30 (0.034)	
Compassion satisfaction	Adaptive cognitive emotion regulation	0.24 (<0.001)	-	0.24 (0.006)	0.50
	Maladaptive cognitive emotion regulation	−0.38 (<0.001)	-	−0.38 (0.006)	
	Occupational stress	−0.15 (0.237)	−0.22 (0.008)	−0.37 (0.055)	
	Experience with aggressive behavior in the workplace	−0.07 (0.626)	−0.18 (0.004)	−0.25 (0.109)	
Compassion fatigue	Adaptive cognitive emotion regulation	−0.06 (0.345)	-	−0.06 (0.407)	0.64
	Maladaptive cognitive emotion regulation	−0.62 (<0.001)	-	0.62 (0.004)	
	Occupational stress	−0.15 (0.237)	0.25 (0.004)	0.17 (0.127)	
	Experience with aggressive behavior in the workplace	−0.07 (0.626)	0.26 (0.004)	0.53 (0.011)	
Adaptive cognitive emotion regulation	Occupational stress	−0.31 (0.051)	-	−0.31 (0.073)	0.18
	Experience with aggressive behavior in the workplace	−0.12 (0.453)	-	−0.12 (0.334)	
Maladaptive cognitive emotion regulation	Occupational stress	0.38 (0.001)	-	0.38 (0.005)	0.57
	Experience with aggressive behavior in the workplace	0.14 (<0.001)	-	0.41 (0.005)	

* SMC = Squared multiple correlations.
